# Investigating the Longitudinal Relationship Between Subjective Cognitive Complaints and Objective Cognition

**DOI:** 10.1093/geroni/igaa057.1988

**Published:** 2020-12-16

**Authors:** Francesca Falzarano, Karen Siedlecki, Jillian Minahan

**Affiliations:** 1 Weill Cornell Medicine, New York, New York, United States; 2 Fordham University, New York, New York, United States; 3 Fordham University, Bronx, New York, United States

## Abstract

Research examining the relationship between subjective cognitive complaints and objective cognitive performance has been mixed. Despite the lack of clear evidence demonstrating an association, subjective cognitive complaints are used as a criterion for the diagnosis of mild cognitive impairment and is considered a risk factor for Alzheimer’s disease. Cross-lagged panel analyses were used in the current study to examine the longitudinal relationships between subjective cognitive complaints (using the Memory Functioning Questionnaire) and objective cognition (e.g., reasoning, memory, spatial visualization, processing speed, and vocabulary) in healthy adults over 60 from the Virginia Cognitive Aging Project (N=441). Results indicated that objective and subjective cognition were only weakly related but that objective cognition is a stronger predictor of subjective cognitive complaints then vice versa. Although subjective cognitive complaints may be an early indicator of pathological aging, results indicate that subjective cognitive complaints may not be a valid predictor of objective cognitive decline.

